# Tools for Implementing an Evidence-Based Approach in Public Health Practice

**DOI:** 10.5888/pcd9.110324

**Published:** 2012-06-21

**Authors:** Julie A. Jacobs, Ellen Jones, Barbara A. Gabella, Bonnie Spring, Ross C. Brownson

**Affiliations:** Author Affiliations: Julie A. Jacobs, Prevention Research Center in St. Louis, Brown School, Washington University in St. Louis, St. Louis, Missouri; Ellen Jones, School of Health Related Professions, University of Mississippi Medical Center, Jackson, Mississippi; Barbara A. Gabella, Colorado Department of Public Health and Environment, Denver, Colorado; Bonnie Spring, Northwestern University Feinberg School of Medicine, Chicago, Illinois.

## Abstract

Increasing disease rates, limited funding, and the ever-growing scientific basis for intervention demand the use of proven strategies to improve population health. Public health practitioners must be ready to implement an evidence-based approach in their work to meet health goals and sustain necessary resources. We researched easily accessible and time-efficient tools for implementing an evidence-based public health (EBPH) approach to improve population health. Several tools have been developed to meet EBPH needs, including free online resources in the following topic areas: training and planning tools, US health surveillance, policy tracking and surveillance, systematic reviews and evidence-based guidelines, economic evaluation, and gray literature. Key elements of EBPH are engaging the community in assessment and decision making; using data and information systems systematically; making decisions on the basis of the best available peer-reviewed evidence (both quantitative and qualitative); applying program-planning frameworks (often based in health-behavior theory); conducting sound evaluation; and disseminating what is learned.

## Introduction

An ever-expanding evidence base, detailing programs and policies that have been scientifically evaluated and proven to work, is available to public health practitioners. The practice of evidence-based public health (EBPH) is an integration of science-based interventions with community preferences for improving population health (1). The concept of EBPH evolved at the same time as discourse on evidence-based practice in the disciplines of medicine, nursing, psychology, and social work. Scholars in these related fields seem to agree that the evidence-based decision-making process integrates 1) best available research evidence, 2) practitioner expertise and other available resources, and 3) the characteristics, needs, values, and preferences of those who will be affected by the intervention (Figure) (2-5).

**Figure:**
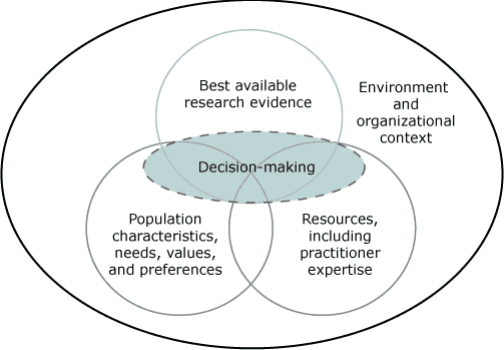
Domains that influence evidence-based decision making. Source: Satterfield JM et al (2).

Public health decision making is a complicated process because of complex inputs and group decision making. Public health evidence often derives from cross-sectional studies and quasi-experimental studies, rather than the so-called "gold standard" of randomized controlled trials often used in clinical medicine. Study designs in public health sometimes lack a comparison group, and the interpretation of study results may have to account for multiple caveats. Public health interventions are seldom a single intervention and often involve large-scale environmental or policy changes that address the needs and balance the preferences of large, often diverse, groups of people.

The formal training of the public health workforce varies more than training in medicine or other clinical disciplines (6). Fewer than half of public health workers have formal training in a public health discipline such as epidemiology or health education (7). No single credential or license certifies a public health practitioner, although voluntary credentialing has begun through the National Board of Public Health Examiners (6). The multidisciplinary approach of public health is often a critical aspect of its successes, but this high level of heterogeneity also means that multiple perspectives must be considered in the decision-making process.

Despite the benefits and efficiencies associated with evidence-based programs or policies, many public health interventions are implemented on the basis of political or media pressures, anecdotal evidence, or "the way it's always been done" (8,9). Barriers such as lack of funding, skilled personnel, incentives, and time, along with limited buy-in from leadership and elected officials, impede the practice of EBPH (8-12). The wide-scale implementation of EBPH requires not only a workforce that understands and can implement EBPH efficiently but also sustained support from health department leaders, practitioners, and policy makers.

## The Need for Evidence-Based Public Health

Calls for practitioners to include the concepts of EBPH in their work are increasing as the United States embarks upon the 10-year national agenda for health goals and objectives that constitutes the *Healthy People 2020* initiative. The very mission of *Healthy People 2020* asks for multisectoral action "to strengthen policies and improve practices that are driven by the best available evidence and knowledge" (13).

Funders, especially federal agencies, often require programs to be evidence based. The American Recovery and Reinvestment Act of 2009 allocated $650 million to "carry out evidence-based clinical and community-based prevention and wellness strategies . . . that deliver specific, measurable health outcomes that address chronic disease rates" (14). The Patient Protection and Affordable Care Act of 2010 mentions "evidence-based" 13 times in Title IV, Prevention of Chronic Disease and Improving Public Health, and will provide $900 million in funding to 75 communities during 5 years through Community Transformation Grants (15).

Federal funding in states, cities, and tribes, and in both urban and rural areas, creates an expectation for EBPH at all levels of practice. Because formal public health training in the workforce is lacking (7), on-the-job training and skills development are needed. The need may be even greater in local health departments, where practitioners may be less aware of and slower to adopt evidence-based guidelines than state practitioners (16) and where training resources may be more limited.

*Core Competencies for Public Health Professionals* (17) emerged on the basis of recommendations of the Institute of Medicine's 1988 report *The Future of the Public's Health*. Last updated in May 2010, these 74 competencies represent a "set of skills desirable for the broad practice of public health," and they are compatible with the skills needed for EBPH (3). Elements of state chronic disease programs and competencies endorsed by the National Association of Chronic Disease Directors are also compatible with EBPH (18).

In addition to efforts to establish competencies and certification for individual practitioners, voluntary accreditation for health departments is now offered through the Public Health Accreditation Board (PHAB). Tribal, state, and local health departments may seek this accreditation to document capacity to deliver the 3 core functions of public health and the Ten Essential Public Health Services (19). One of 12 domains specified by the PHAB as a required level of achievement is "to contribute to and apply the evidence base of public health" (19). This domain emphasizes the importance of the best available evidence and the role of health departments in adding to evidence for promising practices (19).

## Training Programs

Several programs have been developed to meet EBPH training needs, including free, online resources (Box 1).

**Box 1. Selected Tools and Resources for Evidence-Based Public Health (EBPH)**

**Table:** 

**Training tools**
Evidence-Based Public Health (http://prcstl.wustl.edu/EBPH/Pages/Evidence-BasedPublicHealthCourse.aspx). Features slides from the course developed by the Prevention Research Center in St. Louis.
Evidence-Based Behavioral Project Training Portal (www.ebbp.org). Nine modules illustrate the evidence-based practice process for both individual and population-based approaches. Continuing education credits are available for social workers, psychologists, physicians, and nurses.
Evidence-Based Public Health Online Course (http://ebph.ihrp.uic.edu). Produced through the University of Illinois at Chicago's Institute for Health Research and Policy, this online course provides an overview of the EBPH process and includes additional resources and short quizzes.
**Planning tools**
Cancer Control P.L.A.N.E.T. (http://cancercontrolplanet.cancer.gov). The P.L.A.N.E.T. portal walks practitioners through an evidence-based process for cancer control, providing easy access to data and evidence-based resources. Topics include diet/nutrition, physical activity, tobacco control, and more. Step 4 includes practical details on interventions such as time and resources required and suitable settings.
The Community Tool Box (http://ctb.ku.edu). This comprehensive resource offers more than 7,000 pages of practical guidance on a wide range of skills essential for promoting community health. Tool kits (under "Do the Work" tab) provide outlines, examples, and links to tools for topics such as community assessment and evaluation.
Community Health Assessment and Group Evaluation (CHANGE) Tool and Action Guide (www.cdc.gov/healthycommunitiesprogram/tools/change.htm). Developed by the Centers for Disease Control and Prevention (CDC), this tool focuses on assessment and planning. It provides Microsoft Excel (Microsoft, Redmond, Washington) templates for collecting data in 5 sectors: community-at-large, community institutions/organizations, health care, school, and worksite. It is recommended for prioritizing action planning and tracking annual progress in key policy and environmental strategies.
Mobilizing for Action through Planning and Partnerships (MAPP) (www.naccho.org/topics/infrastructure/mapp/index.cfm). The MAPP model, developed by the National Association of County and City Health Officials, guides practitioners through a complete planning process, from beginning organizational steps through assessment and action planning, implementation, and evaluation. The website contains a comprehensive user handbook, a clearinghouse of resources, and stories from the field.
YMCA Community Healthy Living Index (www.ymca.net/communityhealthylivingindex). This site provides assessment tools and planning guides for 6 key community settings: after-school child care sites, early childhood programs, neighborhoods, schools, work sites, and the community at large.
CDC Program Evaluation (www.cdc.gov/eval/index.htm). This site contains step-by-step manuals and other evaluation resources, including the CDC Framework for Program Evaluation.
**US surveillance systems**
Behavioral Risk Factor Surveillance System (BRFSS) (www.cdc.gov/brfss). BRFSS tracks health conditions and risk behaviors annually, using a standard core questionnaire that allows state-specific data to be compared across strata. An interactive menu generates prevalence and trend data by age, sex, race/ethnicity, education, and income level. The SMART (Selected Metropolitan/Micropolitan Area Risk Trends) project provides local data for selected cities and counties.
CDC WONDER (http://wonder.cdc.gov/). CDC WONDER (Wide-ranging Online Data for Epidemiologic Research) provides a single point of access to public health surveillance data and a wide variety of CDC reports, guidelines, and reference materials. Data sets available for query include mortality, natality, cancer incidence, HIV/AIDS, and more.
Youth Risk Behavior Surveillance System (YRBSS) (www.cdc.gov/healthyyouth/yrbs). YRBSS monitors priority health-risk behaviors and the prevalence of obesity and asthma among youth and young adults in the United States.
County Health Rankings (www.countyhealthrankings.org/). Counties in each of the 50 states are ranked according to surveillance data on health outcomes and a broad range of health factors. For each state, data can be downloaded as a Microsoft Excel file; links for relevant state-specific data websites are provided.
**Policy tracking and surveillance**
National Conference of State Legislators (NCSL) (www.ncsl.org/). NCSL provides access to current state and federal legislation and a comprehensive list of state documents, including state statutes, constitutions, legislative audits, and research reports.
Yale Rudd Center for Food Policy and Obesity (www.yaleruddcenter.org/). This site provides a legislation database for federal and state policies on food policy and obesity topics such as breastfeeding, body mass index screenings, and school nutrition.
State Cancer Legislative Database Program (www.scld-nci.net/). The National Cancer Institute maintains this database of state cancer-related health policy.
**Systematic reviews and evidence-based guidelines**
*Guide to Community Preventive Services* (the *Community Guide*) (www.thecommunityguide.org). The Task Force on Community Preventive Services has systematically reviewed more than 200 interventions to produce evidence-based recommendations on population-level interventions. Topics currently include adolescent health, alcohol, asthma, birth defects, cancer, diabetes, health communication, health equity, HIV/AIDS, sexually transmitted infections and pregnancy, mental health, motor vehicle injury, nutrition, obesity, oral health, physical activity, the social environment, tobacco use, vaccines, violence, and worksite health.
The Cochrane Library (www.cochrane.org). More than 5,000 systematic reviews are published in the Cochrane Library, including clinical and population-based interventions and economic evaluations. The Cochrane Public Health Group produces reviews on the effects of population-level interventions (www.ph.cochrane.org).
The Campbell Collaboration (www.campbellcollaboration.org). This international research network produces systematic reviews in education, crime and justice, and social welfare.
**Economic evaluation and gray literature**
Cost-Effectiveness Analysis Registry (https://research.tufts-nemc.org/cear4/home.aspx). This registry offers detailed information on nearly 3,000 cost-effectiveness analyses covering a wide array of diseases and intervention types.
New York Academy of Medicine, Grey Literature Report (www.nyam.org/library/online-resources/grey-literature-report). This bimonthly publication alerts readers to new gray literature on selected public health topics.

In 1997, the Prevention Research Center in St. Louis (PRC-StL) developed an on-site training course, Evidence-Based Public Health. To date, the course has reached more than 1,250 practitioners and has been replicated by PRC-StL faculty in 14 US states and 6 other countries. The course aims to "train the trainer" to extend the reach of the course and build local capacity (Box 2). Course evaluations are positive, and more than 90% of attendees have indicated they will use course information in their work (20-23). Course slides are available online, and a textbook is in its second edition (8). Using a similar framework, the University of Illinois at Chicago developed an online EBPH course that includes short quizzes and additional resources.

**Box 2. Putting Evidence-Based Public Health (EBPH) into Practice**

**Table:** 

**Mississippi**
The Mississippi State Department of Health (MSDH) sponsored an EBPH course, led by faculty from the Prevention Research Center in St. Louis (PRC-StL), for state leaders in July 2010. In April 2011, the course was expanded to local public health districts. At a pre-course workshop, the Southwest District health officer explained the importance of evidence-based community interventions and the role of the health department in community assessment, interventions, and policy. The course itself was taught to 26 local practitioners by instructors from MSDH and PRC-StL. In May 2011, MSDH repeated the course, taught entirely by MSDH staff, in McComb, Mississippi. MSDH included the EBPH model in grant applications to the Coordinated Chronic Disease Program and the Community Transformation Grants program, both initiated by the Centers for Disease Control and Prevention. MSDH offered $15,000 to $26,000 mini-grants to support the development of evidence-based action planning in such areas as physical activity, joint-use agreements, smoke-free municipalities, and healthy corner stores.
**Colorado**
Since May 2011, the Prevention Services Division of the Colorado Department of Public Health and Environment has conducted a pilot project to collaboratively build capacity in EBPH. The 7-step EBPH training approach (3) served as a guide. Epidemiologists and evaluators created practical tools and mini-trainings. One volunteer team focuses on increasing physical activity at the population level while another works to increase screening and referral for pregnancy-related depression during the next 5 years. Both teams completed a community assessment, quantified their health issue, wrote a concise issue statement, rated the evidence on strategies, and prioritized the strategies (steps 1–5). The first team expanded to address obesity prevention and prioritized strategies in April 2012. Division leadership will convene implementation teams to plan and execute the action and evaluation plans for the top-ranked strategies. The team addressing pregnancy-related depression created a logic model using priority strategies, which then informed their state action plan (step 6) that includes SMART (specific, measurable, achievable, relevant, time-bound) objectives and process measures (step 7). At the end of the project in January 2012, this team updated their issue statement and had a portfolio of key documents, tools, and a literature library, intended to sustain capacity in EBPH. This team is implementing the action plan and will semiannually assess the need to repeat any EBPH step.

In 2006, with support from National Institutes of Health, experts from the fields of medicine, nursing, public health, social work, psychology, and library sciences formed the Council for Training in Evidence-Based Behavioral Practice. This group produced a transdisciplinary model of evidence-based practice that facilitates communication and collaboration (Figure) (2,4,5,24) and launched an interactive website to provide web-based training materials and resources to practitioners, researchers, and educators. The EBBP Training Portal, available free with registration, offers 9 modules on both individual and population-based approaches. Users learn how to choose effective interventions, evaluate interventions that are not yet proven, engage in decision making with others, and balance the 3 domains of evidence-based decision making (Figure).

## Key Elements

Key elements of EBPH have been summarized (3) as the following:

- Engaging the community in assessment and decision making;
- Using data and information systems systematically;
- Making decisions on the basis of the best available peer-reviewed evidence (both quantitative and qualitative);
- Applying program planning frameworks (often based in health behavior theory);
- Conducting sound evaluation; and
- Disseminating what is learned.

### Data for community assessment

As a first step in the EBPH process, a community assessment identifies the health and resource needs, concerns, values, and assets of a community. This assessment allows the intervention (a public health program or policy) to be designed and implemented in a way that increases the likelihood of success and maximizes the benefit to the community. The assessment process engages the community and creates a clear, mutual understanding of where things stand at the outset of the partnership and what should be tracked along the way to determine how an intervention contributed to change.

Public health surveillance is a critical tool for understanding a community's health issues. Often conducted through national or statewide initiatives, surveillance involves ongoing systematic collection, analysis, and interpretation of quantitative health data. Various health issues and indicators may be tracked, including deaths, acute illnesses and injuries, chronic illnesses and impairments, birth defects, pregnancy outcomes, risk factors for disease, use of health services, and vaccination coverage. National surveillance sources typically provide state-level data, and county-level data have become more readily available in recent years (Box 1). State health department websites can also be sources of data, particularly for vital statistics and hospital discharge data. Additionally, policy tracking and surveillance systems (Box 1) monitor policy interest and action for various health topics (25).

Other data collection methods can be tailored to describe the particular needs of a community, creating new sources of data rather than relying on existing data. Telephone, mail, online, or face-to-face surveys collect self-reported data from community members. Community audits involve detailed counting of factors such as the number of supermarkets, sidewalks, cigarette butts, or health care facilities. For example, the Active Living Research website (www.activelivingresearch.org) provides a collection of community audit tools designed to assess how built and social environments support physical activity.

Qualitative methods can help create a more complete picture of a community, using words or pictures to describe the "how" and "why"of an issue. Qualitative data collection can take the form of simple observation, interviews, focus groups, photovoice (still or video images that document community conditions), community forums, or listening sessions. Qualitative data analysis involves the verbatim creation of transcripts, the development of data-sorting categories, and iterative sorting and synthesizing of data to develop sets of common concepts or themes (26).

Each of these forms of data collection offers advantages and disadvantages that must be weighed according to the planning team's expertise, time, and budget. No single source of data is best. Most often data from several sources are needed to fully understand a problem and its best potential solutions. Several planning tools are available (Box 1) to help choose and implement a data collection method.

### Selecting evidence

Once health needs are identified through a community assessment, the scientific literature can identify programs and policies that have been effective in addressing those needs. The amount of *available* evidence can be overwhelming; practitioners can identify the *best available* evidence by using tools that synthesize, interpret, and evaluate the literature.

Systematic reviews (Box 1) use explicit methods to locate and critically appraise published literature in a specific field or topic area. The products are reports and recommendations that synthesize and summarize the effectiveness of particular interventions, treatments, or services and often include information about their applicability, costs, and implementation barriers. Evidence-based practice guidelines are based on systematic reviews of research-tested interventions and can help practitioners select interventions for implementation. The *Guide to Community Preventive Services* (the *Community Guide*), conducted by the Task Force on Community Preventive Services, is one of the most useful sets of reviews for public health interventions (27,28). The *Community Guide* evaluates evidence related to community or population-based interventions and is intended to complement the *Guide to Clinical Preventive Services* (systematic reviews of clinical preventive services) (29). 

Not all populations, settings, and health issues are represented in evidence-based guidelines and systematic reviews. Furthermore, there are many types of evidence (eg, randomized controlled trials, cohort studies, qualitative research), and the best type of evidence depends on the question being asked. Not all types of evidence (eg, qualitative research) are equally represented in reviews and guidelines. To find evidence tailored to their own context, practitioners may need to search resources that contain original data and analysis. Peer-reviewed research articles, conference proceedings, and technical reports can be found in PubMed (www.ncbi.nlm.nih.gov/pubmed). Maintained by the National Library of Medicine, PubMed is the largest and most widely available bibliographic database; it covers more than 21 million citations in the biomedical literature. This user-friendly site provides tutorials on topics such as the use of Medical Subject Heading (MeSH) terms. Practitioners can freely access abstracts and some full-text articles; practitioners who do not have journal subscriptions can request reprints from authors directly. Economic evaluations provide powerful evidence for weighing the costs and benefits of an intervention, and the Cost-Effectiveness Analysis Registry tool (Box 1) offers a searchable database and links to PubMed abstracts.

The "gray" literature includes government reports, book chapters, conference proceedings, and other materials not found in PubMed. These sources may provide useful information, although readers should interpret non–peer-reviewed literature carefully. The New York Academy of Medicine produces a bimonthly Grey Literature Report (Box 1), and the US government maintains a website (www.science.gov) that searches the databases and websites of federal agencies in a single query. Internet search engines such as Google Scholar (http://scholar.google.com) may also be useful in finding both peer-reviewed articles and gray literature.

### Program-planning frameworks

Program-planning frameworks provide structure and organization for the planning process. Commonly used models include PRECEDE-PROCEED (30), Intervention Mapping (31), and Mobilizing for Action through Planning and Partnerships (Box 1). Public health interventions grounded in health behavior theory often prove to be more effective than those lacking a theoretical base, because these theories conceptualize the mechanisms that underlie behavior change (32,33). Developed as a free resource for public health practitioners, the National Cancer Institute's guide *Theory at a Glance* concisely summarizes the most commonly used theories, such as the ecological model, the health belief model, and social cognitive theory, and it uses 2 planning models (PRECEDE-PROCEDE and social marketing) to explain how to incorporate theory in program planning, implementation, and evaluation (34). Logic models are an important planning tool, particularly for incorporating the concepts of health-behavior theories. They visually depict the relationship between program activities and their intended short-term objectives and long-term goals. The first 2 chapters of the Community Tool Box explain how to develop logic models, provide overviews of several program-planning models, and include real-world examples (Box 1).

### Evaluation and dissemination

Evaluation answers questions about program needs, implementation, and outcomes (35). Ideally, evaluation begins when a community assessment is initiated and continues across the life of a program to ensure proper implementation. Four basic types of evaluation can achieve program objectives, using both quantitative and qualitative methods. *Formative* evaluation is conducted before program initiation; the goal is to determine whether an element of the intervention (eg, materials, messages) is feasible, appropriate, and meaningful for the target population (36). *Process* evaluation assesses the way a program is being implemented, rather than the effectiveness of that program (36) (eg, counting program attendees and examining how they differ from those not attending).

*Impact* evaluation assesses the extent to which program objectives are being met and may reflect changes in knowledge, attitudes, behavior, or other intermediate outcomes. Ideally, practitioners should use measures that have been tested for validity (the extent to which a measure accurately captures what it is intended to capture) and reliability (the likelihood that the instrument will get the same result time after time) elsewhere. The Behavioral Risk Factor Surveillance System (BRFSS) is the largest telephone health survey in the world, and its website offers a searchable archive of survey questions since the survey's inception in 1984 (Box 1). New survey questions receive a technical review, cognitive testing, and field testing before inclusion. A 2001 review summarized reliability and validity studies of the BRFSS (37).

*Outcome* evaluation provides long-term feedback on changes in health status, morbidity, mortality, or quality of life that can be attributed to an intervention. Because it takes so long to observe effects on health outcomes and because changes in these outcomes are influenced by factors outside the scope of the intervention itself, this type of evaluation benefits from more rigorous forms of quantitative evaluation, such as experimental or quasi-experimental rather than observational study designs.

The Centers for Disease Control and Prevention (CDC) Framework for Program Evaluation, developed in 1999, identifies a 6-step process for summarizing and organizing the essential elements of evaluation (38). The related CDC website (Box 1) maintains links to framework-based materials, step-by-step manuals, and other evaluation resources. Within a detailed outline of the CDC framework's steps, the Community Toolbox also provides tools and examples (Box 1).

After an evaluation, the dissemination of findings is often overlooked, but practitioners have an implied obligation to share results with stakeholders, decision makers, and community members. Often these are people who participated in data collection and can make use of the evaluation findings. Dissemination may take the form of formal written reports, oral presentations, publications in academic journals, or placement of information in newsletters or on websites.

## Putting Evidence to Work

An increasing volume of scientific evidence is now at the fingertips of public health practitioners. Putting this evidence to work can help practitioners meet demands for a systematic approach to public health problem solving that yields measurable outcomes. Practitioners need skills, knowledge, support, and time to implement evidence-based policies and programs. Many tools exist to help efficiently incorporate the best available evidence and strategies into their work. Improvements in population health are most likely when these tools are applied in light of local context, evaluated rigorously, and shared with researchers, practitioners, and other stakeholders.
